# Study on the presence of ochratoxin α in cultures of ochratoxigenic and non- ochratoxigenic strains of *Aspergillus carbonarius*

**DOI:** 10.1371/journal.pone.0185986

**Published:** 2017-10-10

**Authors:** M. Rosa Bragulat, Alba Eustaquio, F. Javier Cabañes

**Affiliations:** 1 Veterinary Mycology Group, Departament of Animal Health and Anatomy, Universitat Autònoma de Barcelona, Bellaterra, Spain; 2 Chemical Analysis Service, Universitat Autònoma de Barcelona, Bellaterra, Spain; Tallinn University of Technology, ESTONIA

## Abstract

Ochratoxin A (OTA) is a potent nephrotoxin and carcinogen which is found in a wide variety of common foods and beverages and it is produced by several species of *Aspergillus* and *Penicillium*. Ochratoxin α (OTα), a major metabolite of OTA, has also been reported to occur in cultures of OTA-producing species. However there is some controversial about the participation of OTα in the biosynthesis of OTA, mainly because its biosynthesis pathway has not yet been completely characterized. *Aspergillus carbonarius* is the main responsible source of ochratoxin A (OTA) in food commodities such as wine, grapes or dried vine fruits from main viticultural regions worldwide. However, little is known about the presence of OTα in isolates of *A*. *carbonarius*. In this study we evaluated the effects of temperature and incubation time on OTα production by both OTA and non-OTA-producing strains of *A*. *carbonarius*. OTA and OTα were detected on the basis of HPLC fluorometric response compared with that of their standards and confirmed by HPLC-MS in selected samples. The non-OTA-producing strains did produce neither OTA nor OTα at any of the conditions tested. The OTA-producing strains studied were able to produce both OTA and OTα in most of the conditions tested. In general, higher amounts of OTA than OTα were produced, but a positive correlation in the production of these two metabolites was detected. The lack of production of both OTA and OTα in the non-OTA-producing strains could be caused by the presence of silent genes or by mutations in functional or regulatory genes involved in OTA production.

## Introduction

Ochratoxin A (OTA) (N-[(3R)-(5-Chloro-8-hydroxy-3-methyl-1-oxo-7-isochromanyl) carbonyl]-L-phenylalanine) is a potent nephrotoxin and carcinogen which is found in a wide variety of common foods and beverages (e.g. cereals, coffee, dried fruits, grape juice, wine). Human exposure to OTA is coming from low level contamination of a wide range of different foods [1]. It was originally described as a metabolite of *Aspergillus ochraceus* and consists of a dihydromethyl-isocoumarin moiety (the polyketide-derived ochratoxin α, OTα) linked to phenylalanine [2]. More recently, many other ochratoxigenic species of *Aspergillus* and also of *Penicillium* have been characterized, although few of them are known to contaminate foods with this mycotoxin. Of these species, *Aspergillus carbonarius* is the main responsible source of OTA in food commodities such as wine, grapes or dried vine fruits from main viticultural regions worldwide [3]. This species is also found in cocoa [4] and coffee beans [5] contaminated by OTA. Besides, OTA production is a very consistent property of this species and for this reason non-OTA-producing isolates of *A*. *carbonarius* are very rarely found in natural environments [6]. However, little is known about the presence of OTα in both OTA and non-OTA-producing isolates of *A*. *carbonarius*.

Although the complete biotransformation pathways of OTA are still unknown, human and animal metabolic studies have shown that OTA is transformed into the less harmful OTα and phenylalanine, among many other OTA-derived metabolites [7, 8]. As well, several enzymes and many species of bacteria and fungi have been reported to be able also to hydrolyze OTA into OTα and phenylalanine [9]. This degradation process is a promising and attractive alternative for the control of OTA in foods and feeds.

On the other hand, OTα has also been reported to occur in cultures of OTA-producing species. However there is some controversial about the participation of OTα in the biosynthesis of OTA, mainly because its biosynthesis pathway has not yet been completely characterized. OTα was suggested to have a strong preferential role in the biosynthesis of OTA using labeled precursors in *A*. *ochraceus* cultures [10]. On the contrary, more recent studies supported the hypothesis that OTα is not involved in the OTA biosynthesis [11, 12]. These studies claim that although OTα is present in cultures of *A*. *carbonarius*, its presence is due to the degradation of OTA.

Nevertheless, in a previous study about other OTA-producing species, *Penicillium verrucosum* mutants were constructed by restriction endonuclease mediated integration [13]. Some of these transformants produced OTα but they were not able to produce OTA. So, in this case OTα was not produced by the hydrolysis of OTA. Possibly, in this study, some of the mutations affect the genes involved in the final step of OTA biosynthesis and OTα was accumulated in the fungal cultures.

Although much information exists concerning the production of OTA at various culture conditions and by different OTA-producing species, there are only a few studies about the production of OTα by these fungi. The effect of temperature among other ecophysiological factors on growth and OTA production on *A*. *carbonarius* has been reported [14], but practically nothing is known about the influence of these factors on OTα production by this species. Besides, there are few studies dealing with wild non-OTA-producing isolates of *A*. *carbonarius* [6, 15]. As nothing is known about the culture conditions for OTα production by this species and it has not been clearly stated its participation in the biosynthesis of OTA, the aim of this study was to evaluate the effects of temperature and incubation time on OTα production by both OTA and non-OTA-producing strains of *A*. *carbonarius*.

## Material and methods

### Fungal isolates

In total, 16 strains of *A*. *carbonarius* from previous grape surveys and different origins were used in this study. The geographical origin and the OTA production ability of the sixteen strains studied are shown in Table 1. Three non-OTA-producing strains (A-2160, A-2579, A-2594) previously reported to be not able to produce OTA [6, 15] were used in this study. The remaining strains were OTA producers and were selected by their different source and different reported ability to produce OTA. They were preserved at -80°C in our culture collection.

**Table 1:** List of *Aspergillus carbonarius* strains from grapes assayed in this study and their properties.

Strain^a^	Geographical origin	OTA production
A-1626	Italy	+
A-1657	France	+
A-1661	France	+
A-1688	Portugal	+
A-1697	Portugal	+
A-1750	Israel	+
A-1759	Israel	+
A-1842	Greece	+
A-1876	Greece	+
A-1998	Australia	+
A-2001	Australia	+
A-2034	Australia	+
A-2071	Spain	+
A-2160	Spain	-
A-2579	Spain	-
A-2594	Spain	-

^a^ Culture collection of the Veterinary Micology group.

### OTA and OTα production ability

OTA and OTα production was detected using a previously described high-pressure liquid chromatography (HPLC) screening method [16] designed in our laboratory for fungi producing OTA in pure culture. Briefly, all the isolates were first three point inoculated on Czapek Yeast extract Agar (CYA) and incubated at 15, 25 and 30°C. After 3, 10 and 30 days of incubation at each temperature assayed and from each isolate, three agar plugs were removed from different points of the colony and extracted with 0.5 ml of methanol. Two additional longer incubation times (60 and 120 days) at 15°C were used for the three non-OTA-producing strains isolates. The extracts were filtered and maintained at 4°C until their analysis. Four replicates for each isolate and incubation condition assayed were used. The entire experiment was repeated twice. In total, eight values of both OTA and OTα for each isolate and incubation condition tested were obtained.

OTA and OTα detection and quantification was made by a Waters 2695 chromatograph with a fluorescence detector Waters 2475 (excitation wavelength: 330 nm/emission wavelength: 460 nm), and with a Sunfire C18 column, 150×4.6 mm, i.d., 3.5 μm. Twenty μl of each extract were applied. The mobile phase was acetonitril/water/acetic acid (57/41/2, v/v/v) eluted at a flow rate of 1 ml/min. The extracts with the same retention time as OTα (around 2.8 min.) and OTA (around 4.8 min), were considered positive, respectively. The limit of quantification of the HPLC technique with the extraction procedure was 0.06 μg/g for OTA and 0.12 μg/g for OTα.

In addition, the identity of OTA and OTα was confirmed in some selected samples by HPLC-MS. A 1200RR HPLC (Agilent Technologies, Waldbronn, Germany) connected to a micrOTOF-Q mass spectrometer (Bruker Daltonics, Bremen, Germany) system was used for the detection of these metabolites. An acetonitril extraction aliquot of each sample was filtered using 0.22μm MS PVDF Syringe Filter from Membrane Solutions (Bellevue, USA), just before injection. The analytes were separated on a 150 x 4.6mm i.d., 3.5 μm, Sunfire column preceded by a 0.5μm guard filter, using an isocratic analysis (20:80, 0.5% HAcO and 1mM NH4AcO in H2O: 0.5% HAcO and 1mM NH4AcO in MeOH) with a flow rate 0.5 mL/min [11]. The column temperature was 25°C, and the injection volume was 20μl. The mass spectrometer was operated in the positive mode, using an electrospray source. The analysis was focused in m/z = 50–1000, using capillary voltage 4800V, nebulizer gas 3.5 Bar, Dry Gas 7.0 L/min, Dry Temp 210°C, Ion Energy 5.0 eV, Collision Energy 7.0 eV, Collision Cell RF 170.0 Vpp, Transfer Time 65 μs and PrePulse Storage Time 8.0 μs. Data acquisition was performed with otofControl version 3.2 and HyStar version 3.2 softwares (Bruker Daltonics, Bremen, Germany) and data processing was performed with Bruker Compass DataAnalysis 4.2 software (Bruker Daltonics, Bremen, Germany). Peak identifications were achieved by comparing retention times and mass spectra of sample peaks with those of standards prepared in acetonitril. All the extracts were injected in a sequence where first and last injection were an OTA plus OTα standard, to verify that the response was stable during the injection samples. A couple of samples without signal for both analytes were spiked with OTA and OTα to verify that there was no suppression signal in sample analysis.

Data obtained from the different conditions tested were statistically analysed by means of one-way analysis of variance test and Student’s test. The Spearman Correlation test was used to correlate the level of production of OTA with that of OTα. All statistical analyses were performed using Minitab 17 statistical software (Minitab Inc, State College, Pennsylvania, USA).

## Results

A total of 16 *A*. *carbonarius* strains were analyzed in order to know if they were able to produce OTA and/or OTα after 3, 10 or 30 days of incubation at 15, 25 and 30°C. Growth was detected in all conditions tested. However poor growth was observed at 15°C after 3 days of incubation.

**Fig 1:**
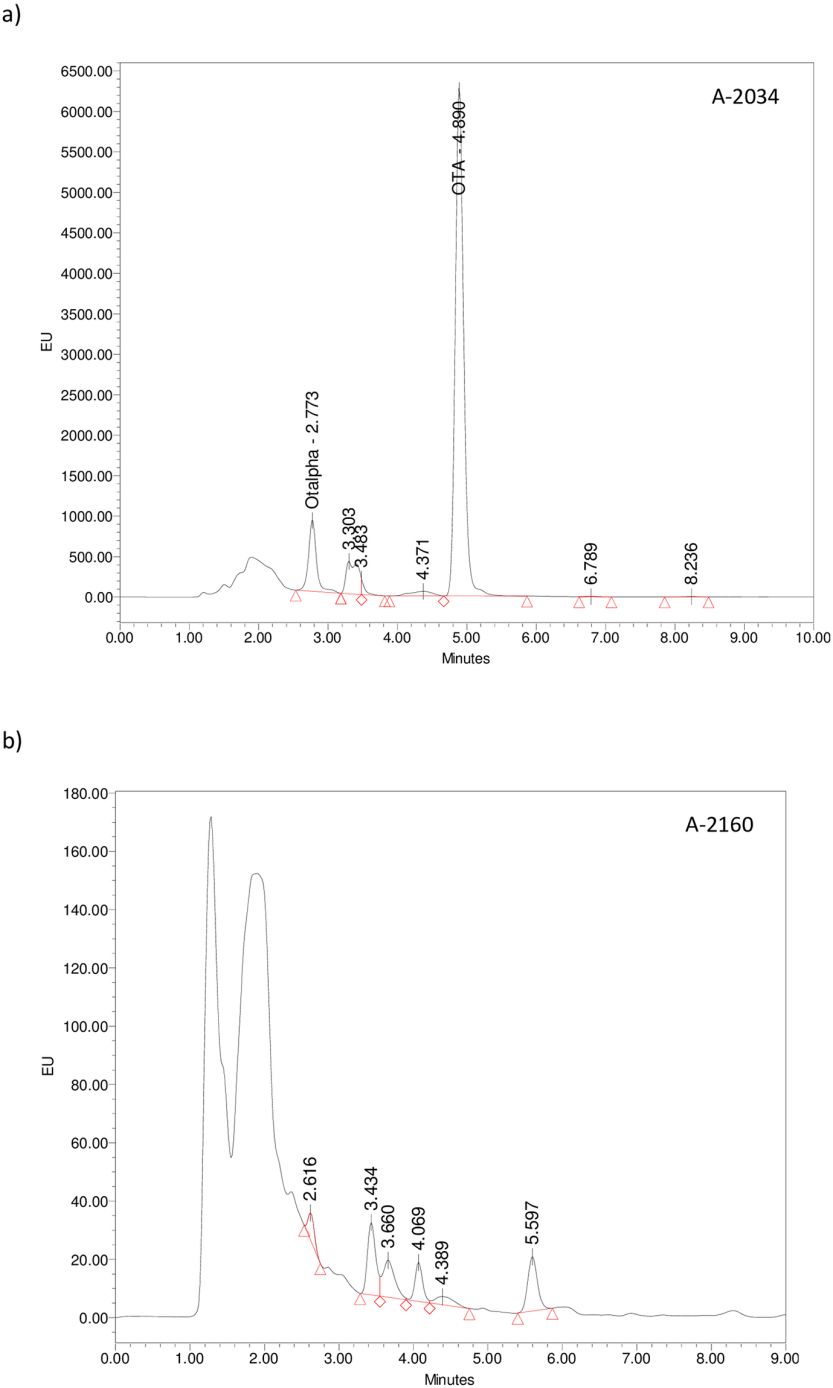
Selected chromatograms of fungal extracts analysed using HPLC coupled to a fluorescence detector of (a) an OTA/OTα-producing strain (*A*. *carbonarius* A-2034) (OTα retention time: 2.773 min; OTA retention time: 4.890 min) and (b) a non-OTA/OTα-producing strain of (*A*. *carbonarius* A-2160), after incubation at 15°C for 30 days on Czapek Yeast extract Agar.

OTA and OTα were quantified on the basis of HPLC fluorometric response compared with that of their standards. Using the method described in this study, we were able to separate these metabolites. Fig 1 shows some selected chromatograms of the fungal strains analysed in this study with HPLC. Extracts of *A*. *carbonarius* A-2034 (Fig 1a) presented a clear peak with the same retention time of OTα (2.773 min) and OTA (4.89 min). The extracts of *A*. *carbonarius* A-2160 (Fig 1b) showed no signals at the same retention time of both OTα and OTA.

**Fig 2:**
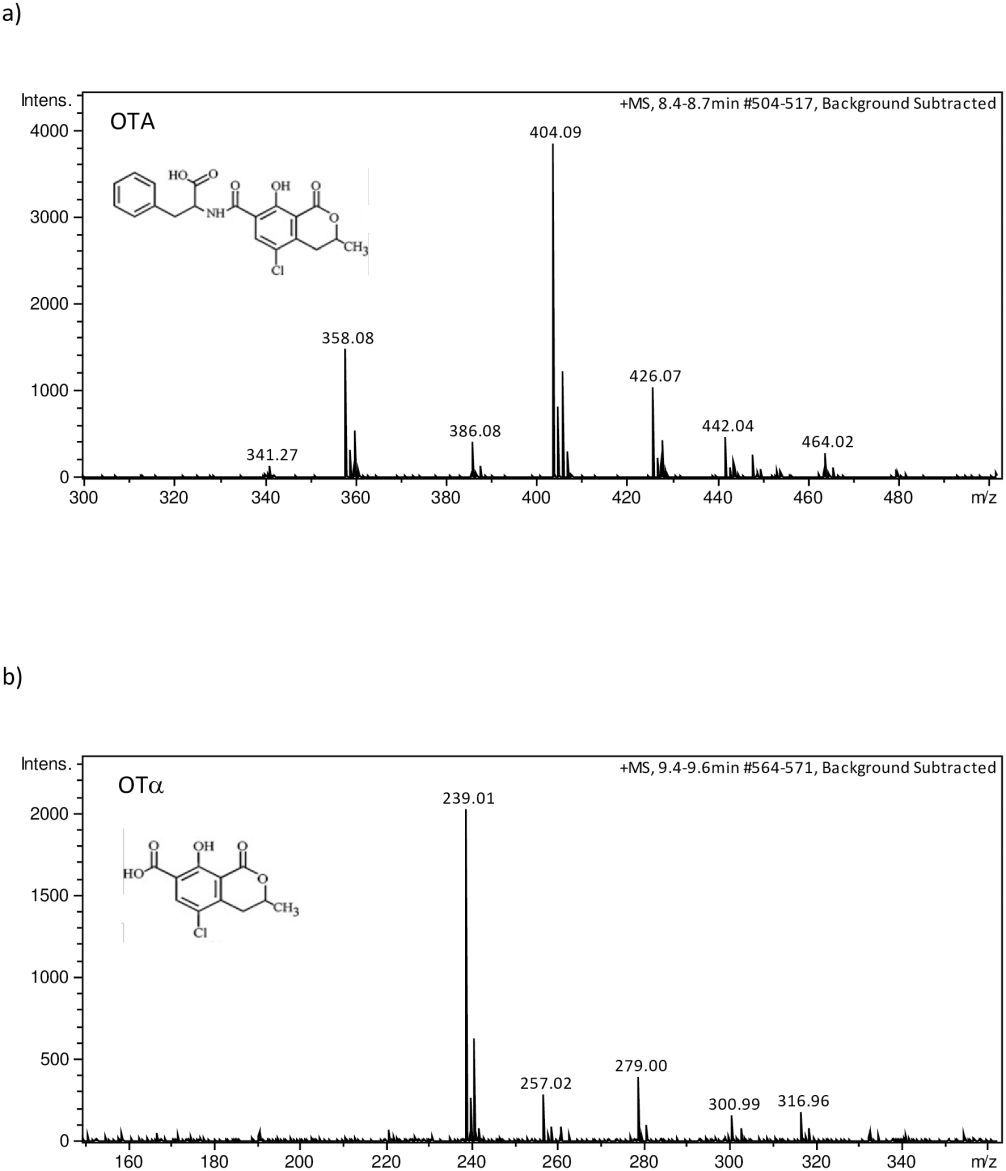
ESI(+)-MS spectra of (a) OTA (major ions: *m*/*z* 358.08 [MH-HCOOH]^+^, *m*/*z* 404.09 [MH]^+^ and *m*/*z* 426.07 [MNa]^+^) and (b) OTα (major ions: *m*/*z* 239.01 [MH-H2O]^+^, *m*/*z* 257.02 [MH]^+^ and *m*/*z* 279.00 [MNa]^+^).

The identity of OTA and OTα was confirmed by HPLC-MS in selected strains at different incubation conditions. OTA was confirmed at 8.5 min by the presence in the MS spectrum of the three major ions with *m*/*z* 358.08 [MH-HCOOH]^+^, *m*/*z* 404.09 [MH]^+^ and *m*/*z* 426.07 [MNa]^+^ similar to those found in the MS spectrum of OTA standard (Fig 2a). OTα was confirmed at 9.5 min by the presence in the MS spectrum of the three major ions with *m*/*z* 239.01 [MH-H2O]^+^, *m*/*z* 257.02 [MH]^+^ and *m*/*z* 279.00 [MNa]^+^ similar to those found in the MS spectrum of OTα standard (Fig 2b).

**Fig 3:**
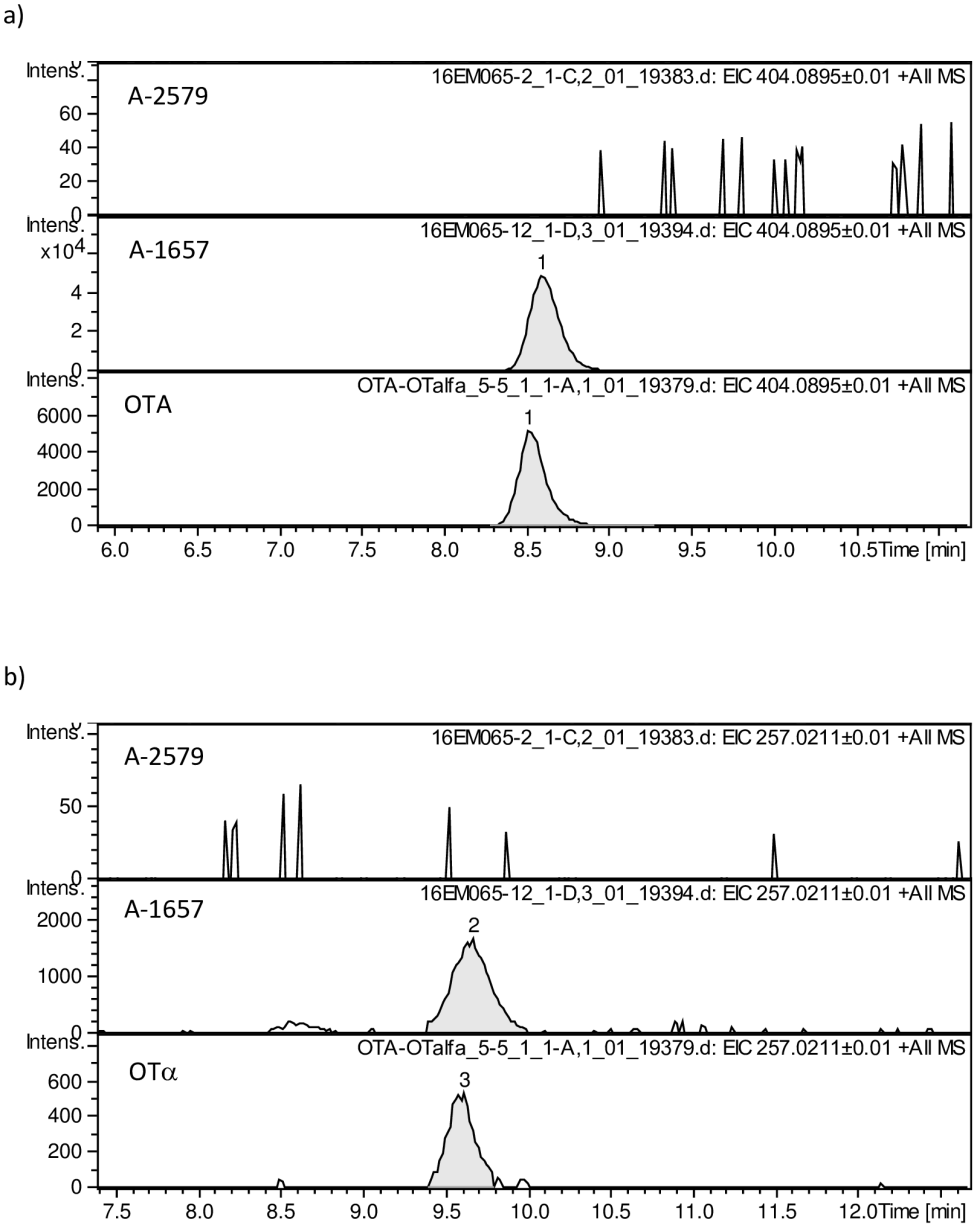
Selected extracted ion chromatograms of fungal extracts and (a) OTA and (b) OTα standards analysed using HPLC-MS and an OTA/OTα-producing strain (*A*. *carbonarius* A-1657) and a non-OTA/OTα-producing strain (*A*. *carbonarius* A-2579), after incubation at 15°C for 30 days on Czapek Yeast extract Agar.

Concentration of OTA and OTα produced by the studied strains in CYA are shown in Tables 2 and 3, respectively. As no statistically significant differences (p > 0.05) were observed between OTA and OTα values recorded in the replicates of each strain, the results are expressed as a mean value. The three confirmed non-OTA-producing strains (A-2160, A-2579, A-2594) did produce neither OTA nor OTα at any of the conditions tested, including the two additional longer incubation times (60 and 120 days) at 15°C. This was confirmed by HPLC-MS. Some selected chromatograms of fungal extracts analysed using HPLC-MS are showed in Fig 3.

**Table 2:** Ochratoxin A (OTA) concentration (mean value in μg/g) detected in the 13 ochratoxigenic strains in CYA at each temperature and incubation time tested.

Ref. strain		OTA (in μg/g)								
	temperature	15°C			25°C			30°C		
	days	3	10	30	3	10	30	3	10	30
A-1626		ND	* ^a^ 31.6 ^a^ †	^b^ 134.1 ^a^	^a^ 5.5 ^b^	^b^ 1.6 ^b^	^b^ 1.8 ^b^	^a^ 1.0 ^c^	^a^ 0.7 ^b^	^a^ 0.8 ^b^
A-1657		ND	^a^ 53.6 ^a^	^b^ 195.2 ^a^	^a^ 30.5 ^b^	^b^ 15.6 ^b^	^b^ 16.2 ^b^	^a^ 17.8 ^c^	^a^ 14.7 ^b^	^a^ 15.4 ^b^
A-1661		ND	^a^ 20.4 ^a^	^b^ 148.4 ^a^	^a^ 14.4 ^b^	^b^ 2.8 ^b^	^b^ 2.8 ^b^	^a^ 4.7 ^c^	^b^ 1.0 ^b^	^b^ 1.0 ^b^
A-1688		ND	^a^ 211.1 ^a^	^a^ 235.9 ^a^	^a^ 22.4 ^b^	^b^ 5.8 ^b^	^b^ 7.9 ^b^	^a^ 7.7 ^c^	^b^ 4.7 ^b^	^b^ 4.2 ^b^
A-1697		ND	^a^ 49.0 ^a^	^b^ 91.4 ^a^	^a^ 4.1 ^b^	^b^ 0.7 ^b^	^b^ 1.2 ^b^	^a^ 0.1 ^a^	^a^ 0.1 ^b^	^a^ 0.4 ^b^
A-1750		ND	^a^ 9.1 ^a^	^b^ 28.8 ^a^	^a^ 10.8 ^b^	^a^ 10.4 ^a^	^a^ 12.5 ^b^	^a^ 11.4 ^b^	^a,b^ 14.8 ^b^	^b^ 18.3 ^c^
A-1759		ND	^a^ 0.8 ^a^	^b^ 11.5 ^a^	^a^ 1.9 ^b^	^b^ 0.4 ^a^	^b^ 0.5 ^b^	^a^ 0.5 ^c^	^a,b^ 0.4 ^a^	^b^ 0.2 ^b^
A-1842		ND	^a^ 100.3 ^a^	^b^ 236.4 ^a^	^a^ 37.6 ^b^	^a^ 33.9 ^a^	^a^ 31.9 ^b^	^a^ 31.5 ^b^	^a^ 35.1 ^a^	^a^ 29.2 ^b^
A-1876		ND	^a^ 26.3 ^a^	^b^ 209.7 ^a^	^a^ 3.7 ^b^	^b^ 0.6 ^b^	^b^ 1.0 ^b^	^a^ 1.0 ^c^	^b^ 0.3 ^b^	^b^ 0.5 ^b^
A-1998		ND	^a^ 38.6 ^a^	^b^ 216.9 ^a^	^a^ 33.0 ^b^	^b^ 10.6 ^b^	^b^ 10.7 ^b^	^a^ 8.2 ^a^	^b^ 2.3 ^b^	^b^ 2.4 ^b^
A-2001		ND	^a^ 7.2 ^a^	^b^ 27.5 ^a^	^a^ 0.5 ^b^	^b^ 0.3 ^b^	^b^ 0.3 ^b^	^a^ 0.3 ^b^	^a^ 0.2 ^b^	^a^ 0.2 ^b^
A-2034		ND	^a^ 23.7 ^a^	^a^ 23.2 ^a^	^a^ 1.0 ^b^	^b^ 0.2 ^b^	^b^ 0.2 ^b^	^a^ 0.3 ^a^	^a,b^ 0.2 ^b^	^b^ 0.2 ^b^
A-2071		ND	^a^ 2.2 ^a^	^b^ 5.4 ^a^	^a^ 0.5 ^b^	^b^ 0.2 ^a^	^b^ 0.2 ^b^	^a^ 0.3 ^c^	^a^ 0.2 ^a^	^a^ 0.2 ^b^
**MEAN**		ND	^a^ 44.1 ^a^	^b^ 120.4 ^a^	^a^ 17.8 ^b^	^a^ 6.4 ^b^	^a^ 6.7 ^b^	^a^ 6.6 ^a,b^	^a^ 5.8 ^b^	^a^ 5.6 ^b^

ND, not detected, Limit of quantification: 0.06 μg/g

* *p value* vs. *time*: In rows, (at the same temperature) mean values not followed by the same letter differ significantly (< 0.05)

^†^ *p value* vs. *temperature*: In rows, (at the same incubation time) mean values not followed by the same letter differ significantly (<0.05)

**Table 3:** Ochratoxin alpha (OTα concentration (mean value in μg/g) detected in the 13 ochratoxigenic strains in CYA at each temperature and incubation time tested.

Ref. strain		OTα (in μg/g)								
	temperature	15°C			25°C			30°C		
	days	3	10	30	3	10	30	3	10	30
A-1626		ND	* ^a^ 5.2 ^a^ †	^a^ 5.1 ^a^	^a^ 0.9 ^b^	^a^ 1.1 ^a,b^	^a^ 1.2 ^b^	^a^ 0.6 ^c^	^a^ 0.7 ^b^	^a^ 0.7 ^c^
A-1657		ND	^a^ 6.1 ^a^	^a^ 5.2 ^a^	^a^ 1.4 ^b^	^b^ 5.2 ^a,b^	^b^ 4.7 ^a^	^a^ 1.3 ^b^	^b^ 4.1 ^b^	^b^ 4.1 ^a^
A-1661		ND	^a^ 2.8 ^a^	^b^ 6.4 ^a^	^a^ 1.0 ^b^	^b^ 1.6 ^b^	^b^ 1.7 ^b^	^a^ 0.5 ^c^	^a^ 0.5 ^c^	^a^ 0.4 ^c^
A-1688		ND	^a^ 8.9 ^a^	^a^ 14.0 ^a^	^a^ 1.3 ^b^	^a,b^ 2.6 ^a^	^b^ 3.4 ^b^	^a^ 1.0 ^c^	^b^ 1.9 ^a^	^b^ 1.8 ^b^
A-1697		ND	^a^ 3.0 ^a^	^a^ 3.9 ^a^	^a^ 0.4 ^b^	^a^ 0.5 ^b^	^a^ 0.5 ^b^	^a^ 0.2 ^c^	^a^ 0.2 ^b^	^a^ 0.2 ^b^
A-1750		ND	^a^ 2.2 ^a^	^b^ 5.3 ^a^	^a^ 1.3 ^b^	^b^ 7.1 ^b^	^b^ 7.3 ^b^	^a^ 2.6 ^c^	^b^ 5.4 ^b^	^b^ 5.9 ^a^
A-1759		ND	^a^ 0.2 ^a^	^b^ 3.0 ^a^	^a^ 0.5 ^b^	^a^ 0.4 ^a^	^a^ 0.5 ^b^	^a^ 0.4 ^c^	^a^ 0.5 ^a^	^a^ 0.3 ^b^
A-1842		ND	^a^ 4.5 ^a^	^b^ 2.9 ^a^	^a^ 1.7 ^b^	^b^ 7.8 ^b^	^b^ 7.0 ^b^	^a^ 1.5 ^b^	^b^ 6.9 ^a,b^	^b^ 5.9 ^b^
A-1876		ND	^a^ 4.7 ^a^	^b^ 7.1 ^a^	^a^ 0.5 ^b^	^b^ 0.8 ^b^	^b^ 0.8 ^b^	^a^ 0.4 ^b^	^a^ 0.4 ^b^	^a^ 0.4 ^b^
A-1998		ND	^a^ 3.2 ^a^	^b^ 10.1 ^a^	^a^ 2.1 ^b^	^b^ 3.7 ^a^	^b^ 3.8 ^b^	^a^ 0.4 ^c^	^b^ 1.4 ^b^	^b^ 1.3 ^c^
A-2001		ND	^a^ 1.2 ^a^	^b^ 6.6 ^a^	^a^ 0.4 ^b^	^a^ 0.4 ^b^	^a^ 0.3 ^b^	^a^ 0.4 ^b^	^b^ 0.2 ^b^	^b^ 0.2 ^b^
A-2034		ND	^a^ 4.6 ^a^	^a^ 6.3 ^a^	^a^ 0.4 ^b^	^a^ 0.3 ^b^	^a^ 0.3 ^b^	^a^ 0.4 ^b^	^b^ 0.2 ^b^	^b^ 0.2 ^b^
A-2071		ND	^a^ 0.3 ^a^	^b^ 3.0 ^a^	^a^ 0.3 ^b^	^b^ 0.5 ^a^	^b^ 0.3 ^b^	^a^ 0.3 ^b^	^a^ 0.2 ^a^	^a^ 0.2 ^b^
**MEAN**		ND	^a^ 3.6 ^a^	^b^ 6.1 ^a^	^a^ 0.9 ^b^	^a^ 2.5 ^a^	^a^ 2.5 ^b^	^a^ 0.8 ^b^	^a^ 1.7 ^a^	^a^ 1.7 ^b^

ND, not detected, Limit of quantification: 0.12 μg/g

* *p value* vs. *time*: In rows, (at the same temperature) mean values not followed by the same letter differ significantly (< 0.05)

^†^ *p value* vs. *temperature*: In rows, (at the same incubation time) mean values not followed by the same letter differ significantly (< 0.05)

The remaining thirteen strains studied were able to produce both OTA and OTα in most of the conditions tested. However, both metabolites were not detected at 15°C after 3 days of incubation, due to the poor growth presented by the strains at this condition. On the contrary, OTA and OTα were early detected at 25°C and 30°C. At these incubation temperatures, all these strains had good growth after 3 days of incubation and in most cases OTA and OTα were clearly detected. However, higher amounts of OTA than OTα were detected in most of the conditions tested.

In these conditions, these strains produced from 0.5 to 37.6 μg/g of OTA (mean concentration: 17.8 μg/g) at 25°C and from 0.1 to 31.5 μg/g of OTA (mean concentration: 6.7 μg/g) at 30°C. However, most strains produced higher amounts of OTA at 15°C after 10 or 30 days of incubation than in the rest of the temperatures and incubation times tested. At 15°C, these 13 strains produced from 0.8 to 211.1 μg/g of OTA (mean concentration: 44.1 μg/g) after 10 days of incubation and from 5.4 to 236.4 μg/g of OTA (mean concentration: 120.4 μg/g) after 30 days of incubation. These strains produced lower amounts of OTA in all the incubation times at 25°C (mean concentration range: 6.4–17.8 μg/g) and at 30°C (mean concentration range: 5.6–6.6 μg/g).

**Fig 4:**
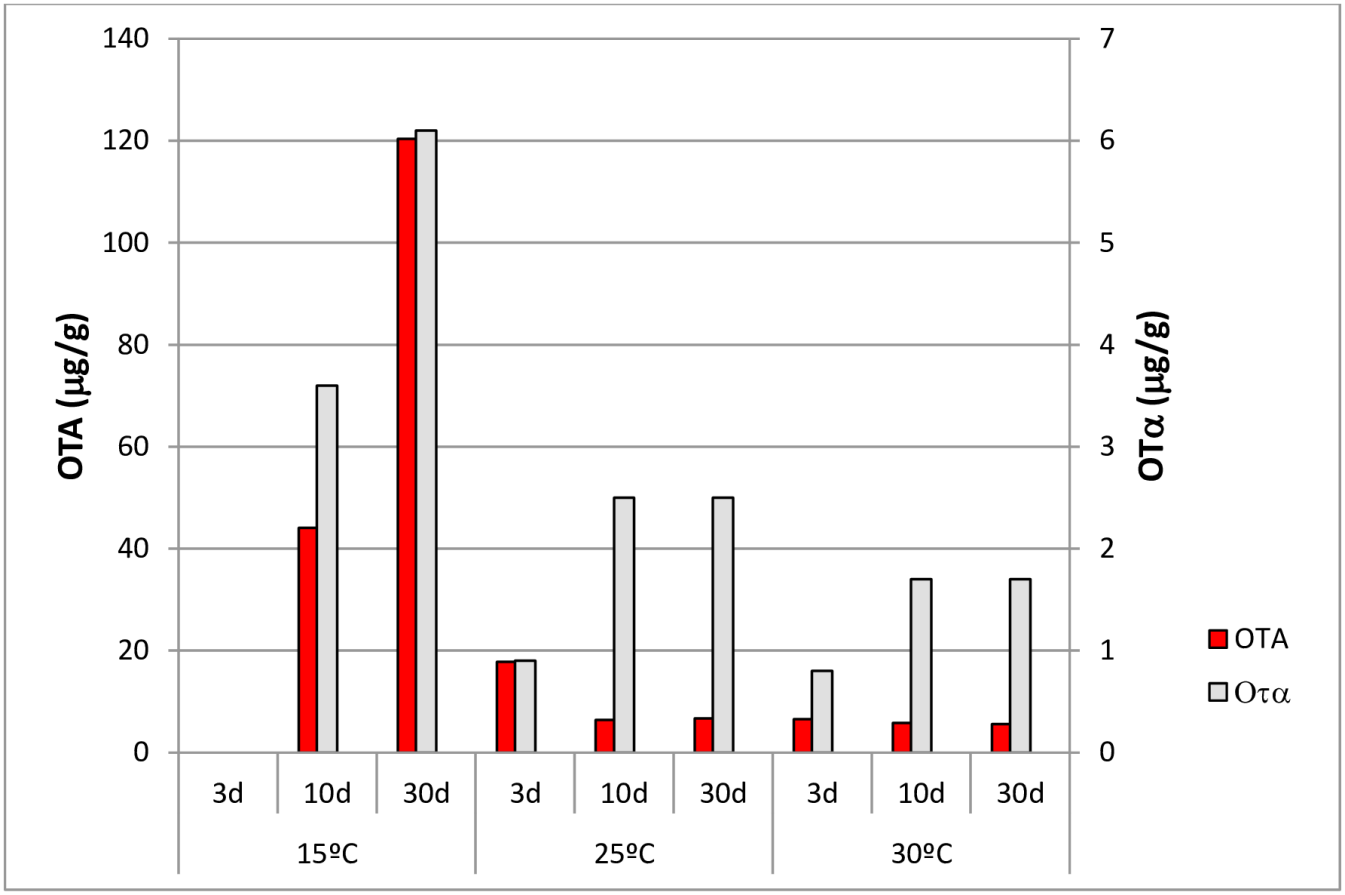
Mean OTA and OTα concentrations produced by the thirteen OTA-producing strains at each temperature and incubation time tested.

As far as OTα production is concerned, different production profiles among the OTA-producing strains were found. However, most strains produced higher amounts of OTα at 15°C after 10 or 30 days of incubation than in the rest of the temperatures and incubation times tested. At 15°C, these thirteen strains produced from 0.2 to 8.9 μg/g of OTα (mean concentration: 3.6 μg/g) after 10 days of incubation and from 2.9 to 6.1 μg/g of OTα (mean concentration: 6.1 μg/g) after 30 days of incubation. These strains produced lower amounts of OTα in all the incubation times at 25°C (mean concentration range: 0.9–2.5 μg/g) and at 30°C (mean concentration range: 0.8–1.7 μg/g) (Fig 4). However, a positive correlation between OTA and OTα production was detected at all incubation temperatures tested. The Spearman correlation coefficients showed that the presence of OTA was positively correlated with the presence of OTα at 15°C (ρ = 0.893), 25°C (ρ = 0.823) and 30°C (ρ = 0.902) (p < 0.05).

## Discussion

The effect of temperature on growth and OTA production on *A*. *carbonarius* has been widely studied [14]. OTA production by *A*. *carbonarius* are usually analyzed after at least 7 days of incubation, but in some studies this toxin has been detected before at different incubation temperatures and culture media. In this species, OTA detection has been reported after 5 days of incubation in CYA at 20°C [17] and after 4 days of incubation at 28°C in PDA plates [18].

Nevertheless, this metabolite has been detected only after 48h of incubation in CYA at 30°C [19] and in Czapek Yeast Extract broth at 25°C [20]. However, nothing is known about the kinetics of OTα production by *A*. *carbonarius*. As far as, the choice of the culture medium used in our study is concerned, CYA medium is reported to be more suitable than YES for OTA production by *A*. *carbonarius* [16, 17]. Consequently, CYA was considered as a proper culture medium also for OTα.

In the present study, various OTA and OTα production profiles among the OTA-producing strains were found. In general, higher amounts of OTA than OTα were detected in most of the conditions tested. We could not detect OTA after 3 days of incubation at 15°C due to the poor growth observed at this temperature. However, most strains produced the higher amounts of both OTA and OTα at 15°C after 10 or 30 days of incubation than in the rest of the temperatures and incubation times tested. As a general trend OTA content increased over incubation time at 15°C. Although optimum conditions for OTA production varied with strain, our results are in accordance with previous studies [17, 21] where *A*. *carbonarius* tend to have the optimal temperatures for OTA production around 15–20°C. This trend was not detected at 25 and 30°C. At these last conditions, OTA content diminished or kept stable during incubation times. Nevertheless, a positive correlation between OTA and OTα production was detected at all incubation temperatures tested.

As we have already mentioned in the introduction of this paper, there is some controversial about the participation of OTα in the biosynthesis of OTA. On the one hand, some authors claim [11, 12] that although OTα is present in cultures of *A*. *carbonarius*, its presence is due to the degradation of OTA. So, its absence could be linked to the loss of production of OTA. On the other hand, in some studies, OTα has been suggested to have a strong preferential role in the biosynthesis of OTA [10] or it has been detected in the absence of OTA [13]. Clearly more research is required to solve this controversial issue. However, this is not easy because the OTA biosynthesis pathway has not yet been completely characterized.

Besides, none of the confirmed non-OTA-producing strains were able to produce OTA or OTα at the conditions tested, including the two additional longer incubation times (60 and 120 days) at 15°C (optimal temperature condition). This was also confirmed by HPLC-MS in the present study. As described in detail previously [6], OTA production is a very consistent property of *A*. *carbonarius* and for this reason atoxigenic isolates of this species are very rarely found in natural environments. Atoxigenic strains of *A*. *carbonarius* could be useful as biotechnological agents to be used in food industry and as biological agents for control of OTA production in vineyards and other crops. The lack of production of both OTA and OTα in these strains could be caused by the presence of silent genes or by mutations in functional or regulatory genes involved in OTA production [22].

Recently, some of us and other colleagues used the Ion Torrent technology to resequence the genome of the non-OTA-producing strain *A*. *carbonarius* A-2160 [15], also evaluated in the present study. As described in full previously [15], we detected that in this strain there was a high accumulation of nonsense and missense mutations in PKS and NRPS encoding genes. Interestingly, a two fold increase in gene mutation ratio was observed in PKS and NRPS encoding genes which are suggested to be involved in OTA biosynthesis. Mutations in these genes, among other causes, could explain the lack of production of both OTA and OTα by these three non-OTA-producing strains.

In order to contribute to elucidate the little-known OTA biosynthetic gene cluster of *A*. *carbonarius*, we plan to investigate the role of the mutations detected in PKS and NRPS encoding genes in these three non-OTA/OTα-producing strains. A full characterization of the gene clusters responsible for OTA production in these strains will show whether they have the gene cluster required for OTA production. In the same direction, we plan also further metabolomic studies of both toxigenic and atoxigenic strains.
